# Chronic inhalation of cigarette smoke reduces phagocytosis in peripheral blood leukocytes

**DOI:** 10.1186/s13104-015-1706-7

**Published:** 2015-11-23

**Authors:** Thomas Tschernig, Andrea Rabung, Meike Voss, Carola Meier, Robert Bals, Christoph Beisswenger

**Affiliations:** Medical Faculty, Institute of Anatomy and Cell Biology, Saarland University, Kirrberger Straße, 66421 Homburg, Saar Germany; Department of Internal Medicine V-Pulmonology, Allergology and Respiratory Critical Care Medicine, Medical Faculty, Saarland University, 66421 Homburg, Saar Germany

## Abstract

**Background:**

Phagocytosis activity of peripheral blood leukocytes in smokers or chronic obstructive pulmonary disease patients was found to be controversial and dependent on the phagocytic stimulus.

**Results:**

We demonstrated that long-term exposure to cigarette smoke in mice clearly suppressed the phagocytosis of granulocytes and monocytes from peripheral blood.

**Conclusions:**

Impaired phagocytosis activity of peripheral blood leukocytes may have a systemic effect and potentially contribute to smoking-associated diseases such as pneumonia and lung cancer.

## Findings

Phagocytic activity in chronic obstructive pulmonary disease (COPD) patients has been well investigated in monocyte-derived macrophages and in alveolar macrophages, as summarized by Louise Donnelly and Peter Barnes [1]. Less research has been carried out on circulating phagocytes. Phagocytic activity of circulating phagocytes, neutrophils and monocytes, is involved both in inflammation as well as in the abatement of inflammation and tissue repair in all organs. Various studies (cited in [1]) established that the phagocytic activity of peripheral blood leukocytes (PBL) in patients with COPD was neither affected nor attenuated by the implementation of a Candida species as a phagocytic stimulus. The PBL were only found to be impaired in one study in which *Escherichia coli* was applied as a phagocytic stimulus [2].

**Fig. 1:**
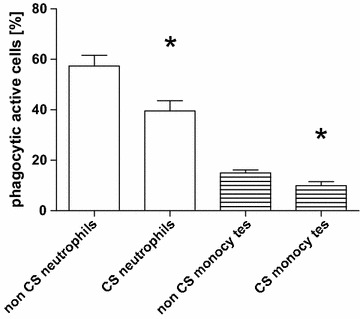
Reduction of active phagocytic cells in the monocytes (*open bars*) and in the neutrophils (*striped bars*) is shown as mean and standard error of the mean. The differences between non-CS and CS exposure were significant with p < 0.05 (ANOVA and Mann–Whitney)

The mechanism through which cigarette smoke (CS) influences circulating phagocytes is unclear. It is also unclear whether impaired activity of circulating phagocytes affects the host defense, this question is however not addressed in this report. We recently presented a mouse model of COPD [3] and showed that phagocytosis—measured as increased mean fluorescence intensity—was suppressed in PBL after long-term exposure to CS [4]. We have now determined the phagocytosis activity of PBL obtained from mice exposed to air or CS for 6 months. The animal experiments were approved by the ethical committee of the Saarland and by the Landesamt für Soziales, Gesundheit und Verbraucherschutz of the Government of Saarland. The mice were maintained in pathogen-free conditions. Briefly, 7–9 week-old female wild-type C57BL/6N mice were exposed to a combination of sidestream and mainstream CS (3R4F, College of Agriculture, Reference Cigarette Program, University of Kentucky, Lexington, Kentucky, USA) in a TE-10 smoking machine (Teague Enterprises, Woodland, California, USA) for a total of 261 min/day, 5 days per week. The smoking time was 87 min with 40 min air exposure in between CS exposures, with three CS exposures/day. Mice were exposed to CS or air for 6 months. This long exposure time of the mice was chosen to simulate the situation in humans with the development of COPD decades later. The CS concentration was 120 mg/m^3^ total suspended particles. Phagocytosis activity of whole blood monocytes and granulocytes was determined using a flow cytometry-based assay (Phagotest, Orpegen Pharma, Germany), according to the instructions. Samples were analyzed in a FACS Calibur flow cytometer (Beckton Dickinson, Heidelberg, Germany). Gates (forward/side scatter) were set on granulocytes and monocytes. For determination of the phagocytosis activity in each sample phagocytosis was, in accordance with the manufacturer’s instructions, determined as a percentage of cells which had ingested fluorescein isothiocyanate (FITC)-conjugated and opsonized *Escherichia coli*. Ice-cold controls were used to avoid simple adhesion. The percentage of active phagocytic cells was significantly reduced in neutrophils (clean air exposure vs. CS exposure: percentage 57 ± 4 vs. 40 ± 4) and monocytes (15 ± 1 vs. 10 ± 2) after CS inhalation (Fig. 1) (N = 10, ANOVA and Mann–Whitney, p < 0.05). A pathological significance of this 30 % reduction in phagocytosis is speculative and has to be evaluated in further studies. Our findings support the hypothesis that inhaled CS has a systemic effect on circulating phagocytes. This could possibly open the door for the investigation of related open questions, mechanisms and therapies in a mouse model of COPD.

## Abbreviations

COPD: chronic obstructive pulmonary disease
CS: cigarette smoke
FITC: fluorescein isothiocyanate

## References

[1] Donnelly LE, Barnes PJ (2012). Defective phagocytosis in airways disease. Chest.

[2] Prieto A, Reyes E, Bernstein ED, Martinez B, Monserrat J, Izquierdo JL, Callol L, de LUCAS P, Alvarez-Sala R, Alvarez-Sala JL, Villarrubia VG, Alvarez-Mon M (2001). Defective natural killer and phagocytic activities in chronic obstructive pulmonary disease are restored by glycophosphopeptical (inmunoferón). Am J Respir Crit Care Med.

[3] Herr C, Han G, Li D, Tschernig T, Dinh QT, Beisswenger C, Bals R (2015). Combined exposure to bacteria and cigarette smoke resembles characteristic phenotypes of human COPD in a murine disease model. Exp Toxicol Pathol.

[4] Voss M, Wonnenberg B, Honecker A, Kamyschnikow A, Herr C, Bischoff M, Tschernig T, Bals R, Beisswenger C (2015). Cigarette smoke-promoted acquisition of bacterial pathogens in the upper respiratory tract leads to enhanced inflammation in mice. Respir Res.

